# Postoperative complications and axial length growth after bilateral congenital cataract surgery: eyes with microphthalmos compared to a comparison group

**DOI:** 10.1038/s41433-024-03176-0

**Published:** 2024-06-21

**Authors:** Yiling Jiang, Yinying Zhao, Jun Ni, Fuman Yang, Dandan Wang, Hengli Lian, Yun-e Zhao

**Affiliations:** 1 Eye Hospital and School of Ophthalmology and Optometry, Wenzhou Medical University, Wenzhou, Zhejiang China; 2 National Clinical Research Center for Ocular Diseases, Wenzhou Medical University, Wenzhou, Zhejiang China; 3 Eye Hospital of Wenzhou Medical University, Hangzhou Branch, Hangzhou, Zhejiang China

**Keywords:** Lens diseases, Eye diseases

## Abstract

**Purpose:**

To investigate the postoperative clinical outcomes and axial length (AL) growth of infants with congenital cataracts and microphthalmos following first-stage cataract surgery.

**Design:**

Retrospective case-control study.

**Methods:**

Setting: Single centre. Infants with congenital cataract that met the inclusion criteria were classified into two groups: the microphthalmos and comparison groups. All infants underwent a thorough ophthalmologic examination before surgery, and one week, 1 month, 3 months, and every 3 months after surgery.

**Results:**

This study enrolled 21 infants (42 eyes) in the microphthalmos group and 29 infants (58 eyes) in the comparison group. More glaucoma-related adverse events were observed in the microphthalmos group (7 eyes, 16.7%) than in the comparison group (0 eyes, 0%) (*p* < 0.001). At each subsequent follow-up, the comparison group had a greater AL than the microphthalmos group (all *p* < 0.001), and AL growth was significantly higher in the comparison group than in the microphthalmos group (all *p* = 0.035). Visual acuity improvement in the microphthalmos group was similar to that of the comparison group.

**Conclusion:**

Early surgical intervention improves visual function in infants with congenital cataracts and microphthalmos although with a higher incidence of glaucoma-related adverse events. After cataract removal, the AL growth of microphthalmic eyes is slower than that of normally developed eyes.

## Introduction

Microphthalmos is a rare structural eye malfunction resulting from abnormalities in the development of the primary optic vesicle [1]. It has an incidence of 0.2–9 per 10,000 births, affects 3–11% of blind children, and is associated with a high prevalence of congenital cataracts [2–5]. Microphthalmos has been described as eyes with an axial length (AL) 2 standard deviations below the age-adjusted mean [6], typically resulting in an AL below 20.5 mm in adult eyes [7, 8].

In infants with congenital cataracts, early cataract surgery is essential to prevent amblyopia and improve visual functions [9, 10]. Although paediatric cataract surgery techniques and postoperative management have consistently improved in recent years, postoperative complications remain important factors that may obstruct visual rehabilitation [11, 12]. Multiple studies [12–15] have reported the incidence of postoperative complications after congenital cataract surgery. In postoperative glaucoma, Zhang et al. [14]. reported that the incidence of glaucoma-related adverse events was 8.10% during a 4-year follow-up. Wang et al. [15]. observed that the cumulative incidence of suspected and definitive glaucoma was 9.97% during a 5-year follow-up.

For paediatric ophthalmologists, surgeries on infants with microphthalmos can be more challenging because of the crowded anterior segment and the high risk of intraoperative and postoperative complications. In a 37-year longitudinal follow-up, Belitsky et al. [16]. reported that the total glaucoma prevalence was 14.8%, including late- (10.7%) and early-onset (4.1%) glaucoma, and that most patients with late-onset glaucoma had microphthalmos. Although various postoperative complications (glaucoma-related adverse events, visual axis obscuration, and posterior synechiae) have been reported in previous studies [12, 14, 15] which investigated clinical outcomes after congenital cataract surgery, a minority of them [17–20] focused on microphthalmic eyes. On the other hand, different definitions of microphthalmos and complications might have affected the results, and most of them did not include a comparison group [17–20].

AL development is an important factor affecting prognosis, close to selection of an appropriate timing and target refraction of second-stage intraocular lens (IOL) implantation. However, AL growth after first-stage cataract surgery (cataract removal) has been disputed in previous studies [21–25] due to different study populations and follow-up periods. In rare cases of microphthalmos, AL growth has not been well studied, and the comparison between children with and without microphthalmos in the same age group requires further study.

Therefore, we investigated the postoperative clinical outcomes and AL growth of infants with congenital cataracts and microphthalmos following first-stage cataract surgery and compared these results with those of contemporary infants without microphthalmos to evaluate postoperative complications and benefits from surgery. Analysis of the postoperative AL growth in the two groups helped us understand the developmental trends of microphthalmic eyes.

## Methods

### Patients selection

This retrospective study enrolled infants with bilateral congenital cataracts who underwent lensectomy combined with limited anterior vitrectomy without primary IOL implantation at the Eye Hospital of Wenzhou Medical University at Hangzhou, China, between January 2016 and October 2020. This retrospective study was approved by the Institutional Review Board/Ethics Committee of Wenzhou Medical University (Optometry Hospital of Wenzhou Medical University, 2021-051-K-43). This study was conducted in accordance with the tenets of the Declaration of Helsinki. Legal guardians of paediatric patients signed informed consent before their being included in the study.

The inclusion criteria were as follows: (1) bilateral congenital cataract, (2) all surgical procedures performed by the same ophthalmologist (YEZ), (3) age at surgery ≤12 months, and (4) followed up for ≥12 months. The exclusion criteria were as follows: (1) eyes concurrent with glaucoma, uveitis, retinopathy of prematurity, traumatic cataract, aniridia, steroid-induced cataract, posterior persistent fetal vasculature causing stretching of the ciliary process, or tractional retinal detachment before surgery; (2) patients with systemic disorders such as Down’s syndrome, which is independently associated with glaucoma [26] and probably causes growth retardation, and Marfan syndrome; and (3) history of previous ocular surgery.

All patients were classified into two groups: the microphthalmos and comparison groups. Microphthalmos was defined as a total AL at least two standard deviations below the mean age [6, 27], equating to 19.2 mm at 1 year and 20.9 mm at adulthood [28]. The microphthalmos group was further classified into the microcornea group (horizontal corneal diameter <9.0 mm) and the relative normal-size cornea group (horizontal corneal diameter ≥9.0 mm).

### Surgical technique

All surgeries were performed by the same experienced congenital cataract surgeon (Yun-e Zhao) under general anaesthesia using the Accurus or Centurion system (Alcon Laboratories, Inc., Fort Worth, TX, USA) with a cut-rate of 2000 per minute and vacuum of 350 mmHg. Two 0.8-mm precise corneal incisions were created at 10 and 2 O’clock using a MANI ophthalmic knife (MANI, Inc., Tochigi, Japan); lens aspiration was performed after anterior vitrectorhexis with a diameter of about 4.5–5.0 mm, followed by posterior vitrectorhexis about 3–3.5 mm and limited anterior vitrectomy with a 23-gauge vitrector and 23-guage irrigating cannula via two cornea incisions. The incisions were closed using 10–0 absorbable Vicryl sutures (Ethicon, Somerville, NJ, USA). Peripheral iridectomy was performed prophylactically in several cases to decrease the possibility of a pupillary block and inflammatory peripheral anterior synechiae. Without primary IOL implantation, all patients remained aphakic after surgery. The interval between the binocular surgeries was within 7 days.

Postoperative topical treatment included:(1) levofloxacin (0.5%) eye drops (Santen Pharmaceutical Co., Ltd., Japan) four times a day for two weeks,(2) tobramycin dexamethasone eye ointment (tobramycin 0.3%, dexamethasone 0.1%; Alcon Laboratories, Inc., USA) once a day for two weeks and (3) tobramycin dexamethasone eye drops (tobramycin 0.3%, dexamethasone 0.1%; Alcon Laboratories, Inc., USA) four times a day, which were tapered for 4–6 weeks postoperatively. Atropinesulfate ophthalmic gels (atropine 1.0%, Shenyang Xingqi Pharmaceutical Co., Ltd, China) were administered once a day for 1 week and later changed to tropicamide phenylephrine eye drops (Mydrin®-P, Santen Pharmaceutical Co., Ltd, Japan) once a day for one month. Optical correction using aphakic spectacles was prescribed for all patients.

### Primary outcome measurements

All paediatric patients underwent a thorough ophthalmologic examination before surgery, and one week, 1 month, 3 months, and every 3 months after surgery. The examination included slit-lamp bio-microscopy, visual acuity assessment, retinoscopy refraction, dilated fundus examination, A-scan ultrasound examination (Axis Nano, Quantel Medical, Cournon, France), and IOP measurement (iCare, Vantaa, Finland). A-scan ultrasound examination was performed at 3 months and every 6 months after surgery.

#### Ocular biological parameters and demographic features

AL, anterior chamber depth (ACD), and lens thickness (LT) were measured using an contact ultrasonic A-scan (Axis Nano, Quantel Medical, Cournon, France), under intranasal dexmedetomidine sedation [29] for incooperative children. The horizontal corneal diameter (HCD) was measured at the beginning of surgery using callipers. IOP was evaluated using an iCare (Vantaa, Finland) rebound tonometer under intranasal sedation, if not cooperatively. Furthermore, AL, ACD, and LT were measured 10 times, and IOP was measured 6 times for each eye; the mean values were recorded. All the measurements were performed by the same technician.

All surgical videos were reviewed by three experienced congenital cataract doctors (YEZ, YYZ, DDW) to identify preexisting posterior capsule defects (PPCD), which included 3 types of PPCD. Type I showed large defect with sinking cortex in anterior vitreous, type II cluster of fibrotic spots in posterior capsule, and type III with concurrent persistent fetal vasculature (PFV) [30].

#### Visual function assessment

Before surgery, due to the young age (<12 months) and poor cooperation, an objective visual assessment was not possible. In the subjective evaluation, we classified infants’ visual function into central/eccentric, steady/unsteady, and maintained/unmaintained, depending on their ability to fixate on or follow a light source or toys of different colours, and the results were recorded.

After surgery, patients with poor cooperation were assessed in the same manner as described above, and the two results were compared. The Teller acuity card (Stereo Optical Co., Inc., USA) procedure was performed on cooperative patients by the same technician. Visual acuity was recorded at the patient’s last follow-up.

#### Postoperative complications

The main postoperative complications were glaucoma-related adverse events, posterior synechiae, and visual axis opacification (VAO).

According to the IATS [31] and the Childhood Glaucoma Research Network (CGRN) [32], glaucoma-related adverse events were defined as glaucoma plus glaucoma suspect. A study eye was diagnosed as having glaucoma if the IOP was >21 mmHg with one or more of the following anatomical changes: (1) corneal enlargement; (2) asymmetrical progressive myopic shift coupled with enlargement of the corneal diameter and/or AL; (3) increased optic nerve cupping defined as an increase of ≥0.2 in the cup-to-disc ratio; or (4) a surgical procedure was performed for IOP control. A study eye was diagnosed as a glaucoma suspect if there were either (1) recordings of two consecutive IOP measurements > 21 mmHg on different dates after topical corticosteroids had been discontinued without any of the anatomical changes listed above for glaucoma, or (2) glaucoma medication was used to control IOP without any of the anatomical changes listed above.

The existence of posterior synechiae was evaluated using slit-lamp microscopy and defined as adhesion of the posterior iris plane and the margin of the anterior vitrectorhexis or pupil deformation. The VAO examination consisted of retroillumination with slit-lamp microscopy. Any obscuration of the visual axis area requiring an Nd:YAG laser [33] or surgical intervention was recorded as VAO.

### Statistical analysis

All statistical analyses were performed using SPSS version 26.0 (IBM Corp., Armonk, NY, USA). Descriptive statistics were used to analyze individual- and eye-level characteristics. The normality of data distribution was analyzed using the Kolmogorov–Smirnov test, and abnormalities in data distribution were analyzed using non-parametric statistical analyses. Values are expressed as the mean ± standard deviation (SD), range, or median (interquartile range [IQR]). The Mann–Whitney *U*-test, Chi-Square Test, or UNIANOVA was employed to compare the parameters among the different groups. The Chi-square test, Fisher’s exact test or generalized estimating equation (GEE) was used to compare the differences in complication incidence between the different groups. The UNIANOVA or GEE was used to adjust binocular correlation influence. A GEE was used to adjust for the difference in AL and AL increments between the different groups in each postoperative period. Logistic regression analysis was used to analyse potential risk factors for postoperative complications, generating odds ratios (ORs) and associated 95% confidence intervals (CIs). Statistical significance was set at p < 0.05.

## Results

### Demographic features and ocular characteristics

**Fig. 1:**
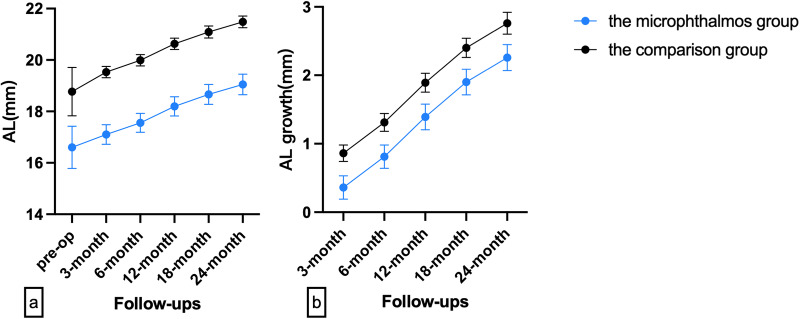
The axial length and its growth of the microphthalmos group (blue dots) and the comparison group (black dots). **a** The axial length. **b** The axial length growth.

This study enrolled 21 infants (42 eyes) in the microphthalmos group and 29 infants (58 eyes) in the comparison group. Demographic features and ocular characteristics of the patients are shown in Table 1. The mean age at surgery of the microphthalmos group was 4.09 ± 1.87 months, and the mean follow-up duration was 34.96 ± 12.65 months. While in the comparison group, the mean age at surgery was 4.84 ± 2.08 months, and the mean follow-up duration was 30.50 ± 9.28 months. The preoperative AL was 16.60 ± 0.82 mm in the microphthalmos group, as compared to 18.77 ± 0.94 mm in the comparison group (Fig. 1). The differences in preoperative AL (*p* < 0.001), LT (*p* = 0.021), HCD (*p* < 0.001), intraoperative dilated pupil size (*p* < 0.001) and last follow-up IOP (*p* < 0.001) between the two groups were statistically significant. Other features, including sex (*p* = 0.416), family history of congenital cataracts (*p* = 0.741), preoperative IOP (*p* = 0.196), mean age at surgery (*p* = 0.068), follow-up duration (*p* = 0.125), and ACD (*p* = 0.376), were similar between the two groups.

**Table 1:** Demographic and ocular features.

	The microphthalmos group	The comparison group	*p*-value
	Individual-level characteristics		
Patients (*n*)	21	29	
Sex (Male/Female)	16/5	19/10	0.416^b^
FHCC (*n*, %)	4, 19.0%	7, 24.1%	0.741^c^
Mean age at surgery (month)	4.09 ± 1.87 (1.7–8.0)	4.84 ± 2.08 (1.2–11.0)	0.068^a^
Follow-up duration (month)	34.96 ± 12.65 (16.0–58.3)	30.50 ± 9.28 (17.9–59.2)	0.125^a^
	Eye-level characteristics		
Eyes (n)	42	58	
Pre-op ocular characteristics			
AL (mm)	16.60 ± 0.82 (15.05–18.35)	18.77 ± 0.94 (16.18–21.10)	<0.001^d^
HCD (mm)	8.75 ± 0.97 (6.5–10.5)	9.77 ± 0.67 (8.2–11.5)	<0.001^d^
LT (mm)	2.86 ± 0.87 (1.56–5.11)	3.47 ± 1.17 (1.76–6.33)	0.021^d^
ACD (mm)	2.35 ± 0.48 (1.81–3.93)	2.48 ± 0.50 (1.83–3.97)	0.376^d^
IOP (mmHg)	10.88 ± 2.42 (6.0−15.0)	11.76 ± 2.34 (6.0−17.0)	0.196^d^
Intra-op dilated pupil size (mm)	5.25 ± 1.09 (2.92–7.66)	6.62 ± 0.89 (4.26–7.85)	<0.001^d^
Last follow-up IOP (mmHg)	15.90 ± 2.74 (12.0–21.0)	14.03 ± 2.24 (6.4–18.5)	<0.001^d^
Microcornea (*n*, %)	21, 50.0%	5, 8.6%	<0.001^b^
PPCD (*n*, %)	24, 57.1%	18, 31.0%	0.009^b^
Pupilary membrane (*n*, %)	14, 33.3%	4, 6.9%	0.001^b^
PFV (*n*, %)	1, 2.4%	4, 6.9%	0.395^c^
Postoperative complications			
Glaucoma-related adverse events (*n*, %)	7, 16.7%	0, 0%	<0.001^e^
Posterior synechiae (*n*,%)	9, 21.4%	11, 19.0%	0.137^e^
VAO (*n*,%)	6, 14.3%	3, 5.2%	0.369^e^
Eyes with postoperative complications (n,%)^†^	16, 38.1%	14, 24.1%	0.646^e^

*FHCC* family history of congenital cataracts, *Pre-op* preoperative, *AL* axial length, *HCD* horizontal corneal diameter, *LT* lens thickness, *ACD* anterior chamber depth *IOP*, intraocular pressure, *Intra-op* intraoperative, *PPCD* preexisting posterior capsular defect, *PFV* persistent fetal vasculature, *VAO* visual axis opacification.

^†^Individually included eyes with glaucoma-related adverse events, posterior synechiae, VAO, and any combination of these three postoperative complications.

^a^Mann–Whitney *U*-test.

^b^Chi-Square test.

^c^Fisher’s exact test.

^d^*P*-value adjusted for binocular correlation influence by UNIANOVA.

^e^*P*-value adjusted for binocular correlation influence by generalized estimating equation.

### Postoperative complications and relative risk factors

There was no significant difference in developing postoperative complications between the microphthalmos group (16 eyes, 38.1%) and the comparison group (14 eyes, 24.1%) (*p* = 0.646). When analyzing specific complications between the groups, more glaucoma-related adverse events were observed in the microphthalmos group (7 eyes, 16.7%; S-Table 1) than in the comparison group (0 eyes, 0.0%) (*p* < 0.001). There was no significant difference in the incidence of other complications: posterior synechiae (9 eyes, 21.4% vs. 11 eyes, 19.0%; *p* = 0.137) or VAO (6 eyes, 14.3% vs. 3 eyes, 5.2%; *p* = 0.369) (Table 1).

In Table 2, the results of the logistic regression analysis were used to evaluate the potential risk factors for postoperative glaucoma-related adverse events in the microphthalmos group. In the univariate analysis, no factor exhibited significant association with glaucoma-related adverse events in the microphthalmos group (all *p* > 0.05), although LT provided an approximate *p*-value (*p* = 0.067).

**Table 2:** Univariate logistic regression analysis of risk factors for glaucoma-related adverse events in the microphthalmos group.

	Univariate logistic regression analysis	
	OR (95% CI)	*p*-value*
Age at surgery	1.263 (0.647–2.466)	0.494
HCD	2.403 (0.571–10.108)	0.232
ACD	1.745 (0.633–4.809)	0.281
LT	6.266 (0.880–44.631)	0.067
AL	8.309 (0.685–100.728)	0.096
Intra-op dilated pupil size	1.841 (0.672–5.045)	0.235

*AL* axial length; *HCD* horizontal corneal diameter; *LT* lens thickness; *ACD* anterior chamber depth; *Intra-op* intraoperative.

^*^P-value adjusted for binocular correlation influence by generalized estimating equation.

### The axial length growth

Excluding cases that developed glaucoma-related adverse events, AL and AL growth were compared between the microphthalmos and comparison groups at 3, 6, 12, 18, and 24 months postoperatively. At each subsequent follow-up, the comparison group had a greater AL than the microphthalmos group (all *p* < 0.001), and AL growth was significantly higher in the comparison group than in the microphthalmos group (all *p* = 0.035) (Fig. 1, S-Table 2).

By the last follow-up, 12 of 35 eyes (excluding 7 eyes that developed glaucoma-related adverse events) in the microphthalmos group had grown to normal AL, while the other 23 eyes remained microphthalmos.

### The visual function

Visual acuity improved in both groups compared to the preoperative conditions as 95 eyes could distinguish thin stripes in the Teller acuity card after surgery (Table 3). At the last follow-up, 27 of 42 eyes (64.3%) in the microphthalmos group gained visual acuity ≥0.5. Despite the comparison group showed a higher proportion, 40 of 58 eyes (69.0%), the two groups showed no significant difference (*p* = 0.623).

**Table 3:** Postoperative decimal visual acuity outcomes*.

	The microphthalmos group (*n* = 42)	The comparison group (*n* = 58)
Unable to cooperate or ≤0.1	5 (11.9%)	0 (0.00%)
0.1	0 (0.00%)	2 (3.4%)
0.15	0 (0.00%)	0 (0.00%)
0.2	0 (0.00%)	0 (0.00%)
0.25	4 (9.5%)	1 (1.7%)
0.3	3 (7.1%)	3 (6.5%)
0.4	3 (7.1%)	12 (20.7%)
0.5	3 (7.1%)	17 (29.3%)
0.6	24 (57.1%)	23 (39.7%)

*Tested using the Teller acuity card procedure.

### Microcornea eyes in the microphthalmos group

The microphthalmic eyes were divided into two subgroups according to the diameter of the cornea: the microcornea group (<9 mm) and the normal cornea group (≥9 mm). Three infants (6 eyes) whose eyes categorized in different groups were excluded. As shown in Table 4, there were no significant differences between the two groups in the preoperative IOP (11.32 ± 2.28 mmHg vs. 10.73 ± 2.62 mmHg; *p* = 0.775), mean age at surgery (3.76 ± 2.02 months vs. 4.06 ± 1.38 months; *p* = 0.323), last follow-up IOP (16.47 ± 2.30 mmHg vs. 15.45 ± 3.09 mmHg; *p* = 0.475), and follow-up duration (39.20 ± 11.58 months vs. 34.53 ± 13.23 months; *p* = 0.152). However, the microcornea group had smaller preoperative AL (16.12 ± 0.65 mm vs. 16.97 ± 0.66 mm; *p* = 0.001), LT (2.50 ± 0.64 mm vs. 3.35 ± 0.97 mm; *p* = 0.017), ACD (2.09 ± 0.22 mm vs. 2.56 ± 0.62 mm; *p* = 0.023) and intraoperative dilated pupil size (4.72 ± 1.02 mm vs. 5.91 ± 0.95 mm; *p* = 0.004) than the normal cornea group (Table 4).

**Table 4:** Demographic and ocular features of the microphthalmos subgroups.

	The microcornea group	The relative normal-size cornea group	*p*-value
	Individual-level characteristics		
Patients (*n*)	9	9	
Mean age at surgery (month)	3.76 ± 2.02 (1.7–8.0)	4.06 ± 1.38 (2.0–6.3)	0.323^a^
Follow-up duration (month)	39.20 ± 11.58 (23.3–58.3)	34.53 ± 13.23 (18.3–55.4)	0.152^a^
	Eye-level characteristics		
Eyes (*n*)	18	18	
Pre-op ocular characteristics			
AL (mm)	16.12 ± 0.65 (15.05-17.24)	16.97 ± 0.66 (15.33–17.89)	0.001^b^
HCD (mm)	7.92 ± 0.73 (6.50–8.90)	9.58 ± 0.51 (9.00–10.50)	<0.001^b^
LT (mm)	2.50 ± 0.64 (1.56–4.04)	3.35 ± 0.97 (1.81–5.11)	0.017^b^
ACD (mm)	2.09 ± 0.22 (1.81–2.55)	2.56 ± 0.62 (1.91–3.93)	0.023^b^
IOP (mmHg)	11.32 ± 2.28 (6.70–15.00)	10.73 ± 2.62 (6.00–13.00)	0.775^b^
Intra-op dilated pupil size (mm)	4.72 ± 1.02 (2.92–6.26)	5.91 ± 0.95 (3.50–7.66)	0.004^b^
Last follow-up IOP (mmHg)	16.47 ± 2.30 (12.10-21.00)	15.45 ± 3.09 (12.00–20.90)	0.475^b^
Postoperative complications			
Glaucoma-related adverse events (*n*, %)	5 (27.8%)	2 (11.1%)	0.923^c^
Posterior synechiae (*n*, %)	5 (27.8%)	2 (11.1%)	0.506^c^
VAO (*n*, %)	5 (27.8%)	1 (5.6%)	0.881^c^
Eyes with postoperative complications (n, %)^†^	9 (50.0%)	5 (27.8%)	0.746^c^

*Pre-op* preoperative, *AL* axial length, *HCD* horizontal corneal diameter, *LT* lens thickness, *ACD*, anterior chamber depth, *IOP* intraocular pressure, *Intra-op* intraoperative, *VAO* visual axis opacification.

^†^Individually included eyes with glaucoma-related adverse events, posterior synechiae, VAO, and any combination of these three postoperative complications.

^a^Mann–Whitney *U*-test.

^b^*P*-value adjusted for binocular correlation influence by UNIANOVA.

^c^*P*-value adjusted for binocular correlation influence by generalized estimating equation.

As for postoperative complications, two groups showed no significant difference in total incidence of the three complications (9 eyes, 50.0% vs. 5 eyes, 27.8%; *p* = 0.746). When each postoperative complication was analyzed separately, no significant differences were observed between the two groups (all *p* > 0.05) (Table 4).

## Discussion

The present study investigated the postoperative complications and axial length growth after bilateral congenital cataract surgery on microphthalmic eyes, and compared these with normally developed ones. The results showed that compared with infants without microphthalmos, infants with contemporary microphthalmos had a higher incidence of glaucoma-related adverse events and a slower AL growth rate, although 12 eyes in the microphthalmos group had grown to normal AL. However, both groups showed considerable improvements in visual acuity.

Glaucoma-related adverse events are vision-threatening complications in patients with congenital cataracts during the postoperative period due to optic nerve damage if not handled promptly and properly. In previous studies, the incidence of glaucoma after paediatric cataract surgery has been reported to vary from 3% to 41% [34, 35]. A few studies [17–20] have reported the incidence of glaucoma in children with congenital cataracts and microphthalmos (S-Table 3). Vasavada et al. [17]. observed that the prevalence of aphakic glaucoma after cataract surgery was 30.9% (13 of 42 eyes), while Prasad et al. [19]. reported a prevalence of 13.5% (5 of 37 eyes). In our study, the incidence of glaucoma-related adverse events was 16.7% in 42 microphthalmic eyes of 21 infants. This discrepancy might reflect differences in the follow-up duration, definition employed, surgical technique, and study populations among studies. Postoperative glaucoma was classified into two types based on its bimodal onset [35, 36]: early-onset closure-angle type and late-onset open-angle type. In our study, two of seven eyes developed postoperative early-onset closure-angle glaucoma, two eyes had late-onset open-angle glaucoma and the other three cases were diagnosed as glaucoma suspects. The mechanism of early-onset glaucoma may be related to genetic predisposing factors [37–40], along with angle closure caused by a pupillary block or inflammatory peripheral anterior synechiae [41–43]. In this study, peripheral iridectomy was performed in two microphthalmic eyes at the primary lens removal procedure, considering the anatomical conditions and risk evaluation; none of the two eyes developed glaucoma. Additionally, Prasad et al. [19]. reported a quite low incidence of glaucoma, 13.5%, with all infants with microphthalmos undergoing peripheral iridectomy during first-stage cataract surgery. We believe that combining peripheral iridectomy with cataract surgery would prevent postoperative glaucoma-related adverse events, highlighting the importance of measuring HCD and careful assessment. Besides, as one patient with an early diagnosis of glaucoma suspect in both eyes was later diagnosed as open-angle glaucoma, reminding us to pay more attention to the long-term follow-up.

Previous long-term large-scale studies [15, 44] revealed potential risk factors for congenital cataract patients after cataract removal, such as microcornea, age at surgery less than 3 months, abnormal anterior segment and etc. Although different risk factors mentioned, the abnormality of eye structure has been quite obvious. The effect of microphthalmos on the risk of developing glaucoma in patients with congenital cataracts is well established [16, 31, 45]. Some studies have further investigated the risk factors for postoperative glaucoma in patients with congenital cataracts and microphthalmos and reported that surgery at an early age and shorter ALs were associated risk factors [17, 19, 20, 31, 36, 40, 46, 47]. However, our results showed that earlier surgical age and shorter AL were not associated with a higher incidence of glaucoma. This could have been because (1) the age at surgery in our study was quite centralized; (2) a few patients had glaucoma; and (3) the follow-up period of this study was relatively limited, which is insufficient for some factors to show their influence. Besides, our study found that smaller dilated pupil size during surgery was nor a risk factor. We supposed that the abnormality of eye structure might be the leading reason for higher postoperative glaucoma incidence in microphthalmos, and in such a cohort it might be hard to find other independent risk factors. Thus, we believe that an extended follow-up period would be valuable for evaluating the long-term incidence of glaucoma, particularly in microphthalmic eyes, and exploring the related risk factors.

A high incidence of inflammatory responses obstructs early visual rehabilitation after paediatric cataract surgery and may cause complex chain reactions. Increased vascular permeability in paediatric patients can easily cause fibrinoid inflammation [48], causing the iris to adhere to the lens capsule membrane, known as posterior synechiae. Severe inflammatory reactions can produce excessive fibrin exudation, forming fibrinous membranes [48]. When the membrane fully covers the pupil area or the posterior synechiae block the fully circumferential pupil, secondary glaucoma associated with pupillary occlusion ensues. The fibrous membrane also contributes to the formation of VAO, and, in the middle and late postoperative stages, another cause is the drastic proliferation and migration of lens epithelial cells [33]. However, adequate anterior vitrectomy could reduce this possibility.

Posterior synechiae were the most frequently observed complication in our study, occurring in 21.4% of 42 microphthalmic eyes, which was lower than previous studies (Praveen et al. [18]. 27.8%; Vasavada et al. [17]. 35.7%). Our team routinely prescribed topical steroids and mydriatic immediately after surgery to prevent posterior synechiae and pupillary occlusion. However, in infants with microcorneas, excessive mydriasis might cause goniosynechia and even angle closing of the anterior chamber, which implies the need for a close follow-up and monitoring by the doctors. Moreover, infants with immature or incompletely mature dilator pupillary muscles cannot fully respond to medication, and some develop posterior synechiae due to stimulation by chronic inflammation. In the present study, the incidence of VAO was 14.3% in the microphthalmos group, which was similar to that in previous studies on microphthalmos, ranging from 5.2% to 16.7% [17, 18, 49, 50]. The incidence of postoperative VAO may also vary according to differences in the individual development of pupil and surgical experience and technique among surgeons.

The visual acuity results of the microphthalmos group in our study were satisfactory and similar to those of the comparison group. Previous studies reported similar results [17, 19, 51]. Despite the increased risk of complications that threaten visual function after surgery, we believe that infants with congenital cataracts and microphthalmos will still benefit from early surgical intervention based on a timely and accurate postoperative optical correction.

This study found that AL growth was slower in the microphthalmos group than in the comparison group after excluding seven eyes with glaucoma-related adverse events. In the study of Sun et al. [21], the growth rate was higher in the microphthalmos group, while there was a significant difference in age at surgery between the two group (3.2 ± 1.3 vs. 5.9 ± 3.0 months, *p* < 0.001), which could affect results. Therefore, our study, with no significant difference in age at surgery between the two groups, might be more convincing. At the last follow-up, 12 eyes (34.3% of 35 eyes) in the microphthalmos group had progressed to age-matched normal AL. AL is related to hereditary factors [52], physical development [49, 53], nutrition, and other environmental factors. We hypothesized that some patients with microphthalmos might suffer more from form deprivation caused by their cataracts, which probably inhibit eyeball development rather than genetic defects. Previous studies built postoperative AL prediction models for children undergoing congenital cataract surgery, based on baseline AL, age at surgery and IOL status [54, 55]. However, the postoperative AL of our microphthalmos patients deviate from these models. One hand, patients with microphthalmos occupy a relatively small proportion in congenital cataracts, leading large error in prediction. The other hand, the mechanism of axial elongation in microphthalmos could be more complex, and future AL growth in microphthalmic eyes remains difficult to predict, which requires a larger sample size and a longer follow-up period for further investigation.

The results of this study should be viewed in light of these limitations. First, it was a retrospective study, this design itself maybe a factor affecting the accuracy of axial length. Despite so, our study maintained the consistency of follow-ups as possible, which, to some extent, avoiding the effect. Second, the sample size was relatively small and the follow-up period was relatively short; a larger sample size and a longer follow-up period may reveal the development of glaucoma, especially the late-onset open-angle type, in some eyes. Further prospective studies with larger sample sizes and longer follow-up periods are warranted.

## Conclusions

In conclusion, early surgical intervention improves visual function in infants with congenital cataracts and microphthalmos. And microphthalmic eyes had a higher incidence of postoperative glaucoma than normally developed eyes. After cataract removal, the AL growth of microphthalmic eyes is slower than that of normally developed eyes, providing an important basis for surgeons to choose the timing of secondary IOL implantation.

## Summary

### What was known before

- Microphthalmos is one of the risk factors of developing glaucoma in patients with congenital cataracts. Early cataract surgery is essential for preventing amblyopia and improving visual function in infants with cataracts.

### What this study adds

- Early surgical intervention improves visual function in infants with congenital cataracts and microphthalmos although with a higher incidence of glaucoma-related adverse events. The axial length growth of microphthalmic eyes is slower than that of normally developed eyes after cataract removal.

## Supplementary information

S-Table 1

S-Table 2

S-Table 3

## Data Availability

The data that support the findings of this study are available from the corresponding author upon reasonable request.
