# Supplementary material for: Postoperative complications and axial length growth after bilateral congenital cataract surgery: eyes with microphthalmos compared to a comparison group
Source: Eye (Lond). 2024 Jun 21;38(15):2912–9. doi: 10.1038/s41433-024-03176-0 (PMC11461963; doi:10.1038/s41433-024-03176-0)
Supplement: Supplementary file 1 — S-Table 1 [file 41433_2024_3176_MOESM1_ESM.docx]

Table 1 Postoperative glaucoma-related adverse events in the microphthalmos group

| Pt | Eye | Age at surgery (month) | Peripheral iridectomy at first-stage | Glaucoma-related adverse events | Management | Follow-up duration  (month) |
| --- | --- | --- | --- | --- | --- | --- |
| 1 | OD | 3.6 | No | Glaucoma suspect | Topical antiglaucoma medication | 55.4 |
|  | OS | 3.8 | No | Glaucoma suspect | Topical antiglaucoma medication | 55.4 |
| 2 | OD | 5.8 | No | Open-angle glaucoma | Topical antiglaucoma medication | 58.2 |
|  | OS | 5.7 | No | Open-angle glaucoma | Topical antiglaucoma medication | 58.3 |
| 3 | OS | 2.1 | No | Glaucoma suspect | Topical antiglaucoma medication | 43.5 |
| 4 | OD | 1.8 | No | Secondary angle-closure glaucoma | Topical antiglaucoma medication+Surgery | 51.5 |
|  | OS | 1.7 | No | Secondary angle-closure glaucoma | Topical antiglaucoma medication+Surgery | 51.7 |
