# Supplementary material for: Postoperative complications and axial length growth after bilateral congenital cataract surgery: eyes with microphthalmos compared to a comparison group
Source: Eye (Lond). 2024 Jun 21;38(15):2912–9. doi: 10.1038/s41433-024-03176-0 (PMC11461963; doi:10.1038/s41433-024-03176-0)
Supplement: Supplementary file 2 — S-Table 2 [file 41433_2024_3176_MOESM2_ESM.docx]

Table 2 Changes in AL (mm) in the two groups

|  | AL (mm) | |  | AL growth (mm) | |  |
| --- | --- | --- | --- | --- | --- | --- |
| Follow-up point | The microphthalmos group | The comparison group | *p*-value | The microphthalmos group | The comparison group | *p*-value |
| Pre-op | 16.60 ± 0.82 | 18.77±0.94 | <0.001^a^ | / | / | / |
| 3-month | 17.10 ± 0.38^b^ | 19.53 ± 0.22^b^ | <0.001^b^ | 0.36 ± 0.17^b^ | 0.86 ± 0.12^b^ | 0.035^b^ |
| 6-month | 17.56 ± 0.37^b^ | 19.99 ± 0.22^b^ | <0.001^b^ | 0.81 ± 0.17^b^ | 1.31 ± 0.13^b^ | 0.035^b^ |
| 12-month | 18.20 ± 0.38^b^ | 20.63 ± 0.22^b^ | <0.001^b^ | 1.39 ± 0.18^b^ | 1.89 ± 0.14^b^ | 0.035^b^ |
| 18-month | 18.66 ± 0.39^b^ | 21.09 ± 0.23^b^ | <0.001^b^ | 1.90 ± 0.19^b^ | 2.40 ± 0.14^b^ | 0.035^b^ |
| 24-month | 19.05 ± 0.40^b^ | 21.48 ± 0.23^b^ | <0.001^b^ | 2.26 ± 0.19^b^ | 2.76 ± 0.16^b^ | 0.035^b^ |

^a^ *p*-value adjusted for binocular correlation influence by UNIANOVA, ^b^ values adjusted for sample size difference at every postoperative follow-up, and binocular correlation influence by generalized estimating equation
