# Supplementary material for: Postoperative complications and axial length growth after bilateral congenital cataract surgery: eyes with microphthalmos compared to a comparison group
Source: Eye (Lond). 2024 Jun 21;38(15):2912–9. doi: 10.1038/s41433-024-03176-0 (PMC11461963; doi:10.1038/s41433-024-03176-0)
Supplement: Supplementary file 3 — S-Table 3 [file 41433_2024_3176_MOESM3_ESM.docx]

Table 3 Studies on infants with microphthalmos following cataract surgery

| Study | Sample size (eye/children) | Definition of microphthalmos | Mean age at surgery (month) | Mean follow-up period (month) | Posterior synechiae | VAO | Glaucoma | Definition of glaucoma | First-stage peripheral iridectomy | population |
| --- | --- | --- | --- | --- | --- | --- | --- | --- | --- | --- |
| Vasavada et al. (2009) | 42 eyes of 21 infants | an AL more than 2 SD smaller than the normal for that age group | 4.0±2.6  (0.5–10.1) | 25.6±11.3 | 35.7% | 16.7% | 30.9% | NA | Not  reported | Indian |
| Praveen et al. (2015) | 72 eys of 36 children | an AL that was 2 SDs smaller than what is normally expected at that age | 4.8±6.2  (0.5–15.0) | 45.80 ± 2.90 | 27.8% | 11.1% | 30.6% | IOP was greater than or equal to 25 mm Hg on more than one occasion. | Not  reported | Indian |
| Prasad et al. (2015) | 37 eyes of 20 infants | AL of the globe is 16.50 mm or less | 3.78±2.25  (0.5–9.0) | 18±5.12 | 13.5% | 10.8% | 13.5% | NA | All infants | Not reported |
| Kim et al. (2019) | 38 eyes of 19 children | a total AL at least 2 SDs below the mean for age | 3.2±1.7  (0.7–6.8) | 93.48 ± 9.04 | Not reported | Not  reported | 29.0% | IOP was ≥26 mm Hg on at least two consecutive visits or clinician’s decision for surgical intervention or permanent medical glaucoma therapy. | Not  reported | Korean |
| The present study | 42 eyes of 21 infants | a total AL at least 2 SDs below the mean for age | 4.09±1.87 (1.7-8.0) | 34.96±12.65 (16.0-58.3) | 21.4% | 14.3% | 16.7% | See **Methods section** | Part of infants | Chinese |

*VAO,* Visual axis opacification; *AL,* axial length; *SD,* standard deviation.
